# Abrogation of greater graft failure risk of female-to-male liver transplantation with donors older than 40 years or graft macrosteatosis greater than 5%

**DOI:** 10.1038/s41598-023-38113-w

**Published:** 2023-08-09

**Authors:** Sangbin Han, Ji Hye Kwon, Kyo Won Lee, Sanghoon Lee, Gyu Sung Choi, Jong Man Kim, Justin Sangwook Ko, Mi Sook Gwak, Gaab Soo Kim, Sang Yun Ha, Jae-Won Joh

**Affiliations:** 1 Department of Anesthesiology and Pain Medicine, Samsung Medical Center, Sungkyunkwan University School of Medicine, Seoul, Korea; 2 Department of Surgery, Samsung Medical Center, Sungkyunkwan University School of Medicine, Seoul, Korea; 3 Department of Pathology and Translational Genomics, Samsung Medical Center, Sungkyunkwan University School of Medicine, Seoul, Korea; 4 Department of Surgery, Samsung Medical Center, Sungkyunkwan University School of Medicine, Changwon, Korea

**Keywords:** Gastroenterology, Risk factors

## Abstract

Greater graft-failure-risk of female-to-male liver transplantation (LT) is thought to be due to acute decrease in hepatic-estrogen-signaling. Our previous research found evidence that female hepatic-estrogen-signaling decreases after 40 years or with macrosteatosis. Thus, we hypothesized that inferiority of female-to-male LT changes according to donor-age and macrosteatosis. We stratified 780 recipients of grafts from living-donors into four subgroups by donor-age and macrosteatosis and compared graft-failure-risk between female-to-male LT and other LTs within each subgroup using Cox model. In recipients with ≤ 40 years non-macrosteatotic donors, graft-failure-risk was significantly greater in female-to-male LT than others (HR 2.03 [1.18–3.49], *P* = 0.011). Within the subgroup of recipients without hepatocellular carcinoma, the inferiority of female-to-male LT became greater (HR 4.75 [2.02–11.21], *P* < 0.001). Despite good graft quality, 1y-graft-failure-probability was 37.9% (23.1%–57.9%) in female-to-male LT within this subgroup while such exceptionally high probability was not shown in any other subgroups even with worse graft quality. When donor was > 40 years or macrosteatotic, graft-failure-risk was not significantly different between female-to-male LT and others (*P* > 0.60). These results were in agreement with the estrogen receptor immunohistochemistry evaluation of donor liver. In conclusion, we found that the inferiority of female-to-male LT was only found when donor was ≤ 40 years and non-macrosteatotic. Abrogation of the inferiority when donor was > 40 years or macrosteatotic suggests the presence of dominant contributors for post-transplant graft-failure other than graft quality/quantity and supports the role of hepatic-estrogen-signaling mismatch on graft-failure after female-to-male LT.

## Introduction

The sex difference in graft failure risk is well-known in liver transplantation (LT). That is, post-transplant graft failure is more prevalent in male recipients with female donors, so-called female-to-male LT, compared to other donor-recipient sex combinations. Since the landmark study by Kahn et al.^1^, clinical studies have consistently demonstrated the inferiority of female-to-male LT^2–7^. Recent research further validated the inferiority of female-to-male LT in living donor LT^8–10^. Our previous research further demonstrated that the inferiority of female-to-male LT is not attributed to anatomical size mismatch between females and males (e.g. relatively small graft size)^11^.

Although the exact mechanisms underlying the sex difference is not fully understood, hepatic estrogen signaling mismatch is considered to play an important role^12–15^. The liver is one of the target organs of estrogen, and hepatic estrogen signaling plays an important role in mitigating injury and enhancing recovery of the liver^10, 12, 16–18^. Hepatic estrogen signaling increases in relation to the increase in systemic estrogen secretion or hepatic estrogen receptor content^19^. In this regard, previous experimental and clinical studies have demonstrated that the tolerance of various liver injuries is greater in females with greater systemic estrogen secretion and hepatic estrogen receptor content compared to males^18, 20, 21^. However, abrupt decrease in hepatic estrogen receptor occurs in the transplanted female liver after exposure to the male hormonal milieu^14^, and consequent decrease in hepatic estrogen signaling and impairment of hepatic protection capacity has been implicated in the greater graft failure risk^6–8^.

In our recent study of healthy living liver donors, we found that the tolerance of hepatic ischemia–reperfusion injury is greater in females than in males only when the age is ≤ 40 years (in relation to the decrease in female systemic estrogen secretion after 40 years, which is the age of poor ovarian reserve)^22–25^ and the liver is without macrosteatosis (in relation to the decrease in female hepatic estrogen receptor expression with macrosteatosis)^16, 18, 26, 27^. These findings suggested that female’s superior hepatic protection capacity decreases in relation to the decrease in hepatic estrogen signaling with poor ovarian reserve or macrosteatosis; thus, we deduced that the degree of acute post-transplant defeminization of the graft after female-to-male LT decreases in relation to donor poor ovarian reserve and macrosteatosis. In this study, we aimed to evaluate whether the inferiority of female-to-male LT changes by donor age of 40 years or graft macrosteatosis.

## Patients and methods

### Patients and data sources

We screened the records of 813 recipients who underwent a first adult-to-adult living donor LT between December 2005 and April 2016 (representing the 500th–1700th LT) in our hospital. We excluded 32 recipients of grafts consisting of a partial liver other than the right lobe (extended right lobe, n = 16; left lobe, n = 12; extended left lobe, n = 3; and left lateral segment, n = 1). We further excluded one recipient who underwent an auxiliary partial orthotopic LT. The remaining 780 recipients were included in the analysis. All data analyzed in the study were derived from our institution’s electronic medical records and LT database (prospectively collected). All recipients were followed until September 2017 or the last medical follow-up, for a maximum of 5 years. The Institutional Review Board of Samsung Medical Center approved this retrospective cohort study (SMC 2020-03-015-002) and waived the requirement for written informed consent. The study was conducted in accordance with the principles of the Declaration of Helsinki and Good Clinical Practice Guidelines.

### Liver donation and transplant criteria

The sex of the donor and recipient was not considered in selection of the donor. Acceptance criteria for liver donation were age of ≤ 65 y, body mass index < 35 kg/m^2^, macrosteatosis degree ≤ 30%^28–30^, and donor residual liver volume ≥ 30%. Individuals with any type of hepatitis, including steatohepatitis, or liver fibrosis were excluded from donation.

### Histologic macrosteatosis evaluation

All living donors underwent a wedge liver biopsy before liver resection, and frozen biopsy sections were stained with hematoxylin–eosin and Oil Red O. In general, the size of the biopsy section was 3 μm in thickness, 15 mm in length, and 5 mm in width, containing 15–20 portal fields. Permanent biopsy sections were prepared with tissues being fixed in 4% neutral buffered formalin solution, embedded in paraffin, and stained with hematoxylin–eosin. The pathology report was recorded based on the review process of both frozen and permanent biopsy sections (5–8 serial sections in general), describing the average percentage of macrovesicular fatty droplets occupying the surface area of the parenchyma. For the purpose of the study, macrosteatosis was defined if ≥ 5% hepatocytes contain macrovesicular fat droplets based on a non-alcoholic fatty liver disease activity score system^31, 32^ as well as the study reporting that hepatic estrogen receptor content is lower in females with > 5% macrosteatosis than in females with ≤ 5% macrosteatosis^16^.

### Estrogen receptor evaluation by immunohistochemistry

To examine the mechanisms underlying the specificity of female-to-male LT and its interaction with graft macrosteatosis and donor age, the data of our previous study on immunohistochemistry for estrogen receptor performed for the liver tissues biopsied during the donor hepatectomy were analyzed while 457 patients with available tissues were overlapped between the two studies. The liver tissue was stored in an in-house bio-bank using a a Bond-max autoimmunostainer (Leica Biosystem, Melbourne, Australia) with Bond™ Polymer refined detection, DS9800 (Vision Biosystems, Melbourne, Australia). After deparaffinization of formalin-fixed and paraffin-embedded tissue sections in xylene for a total of 15 min, antigen retrieval was performed at 97 °C for 20 min in ER2 buffer. Briefly, antigen retrieval was performed at 97 °C for 20 min in ER2 buffer. After blocking endogenous peroxidase activity with 3% hydrogen peroxidase for 10 min, slides were incubated with mouse monoclonal estrogen receptor antibody (NCL-L-ER-6F11, Novocastra, Newcastle, United Kingdom) and rabbit monoclonal AR-V7 specific antibody (ab198394, Abcam, San Francisco, CA, USA) for 15 min at room temperature, at a dilution of 1:200. Normal breast tissue was used as a positive control. For ER status evaluation, the Allred score, which is the sum of two scores (percentage of positive hepatocytes showing nuclear staining, range 0–3; intensity of immunoreactivity, range 0–5), was calculated^18, 33^. ER positive expression was defined when the Allred score is greater than or equal to 4 based on previous reserach^34^.

### Operative management

All study donors underwent computed tomography angiography and magnetic resonance cholangiopancreatography to determine the anatomy of the hepatic vascular and biliary system. Expert liver radiologists and transplant surgeons carefully discussed anatomical considerations and related surgical issues in a joint meeting prior to transplantation. All grafts consisted of segment 5 through 8, excluding the middle hepatic vein trunk. Graft implantation was performed using the piggyback technique. After the portal vein anastomosis was complete, the graft was reperfused by consecutively unclamping the hepatic vein and portal vein. Subsequently, hepatic artery anastomosis was followed by biliary anastomosis. Transfusion of allogeneic blood was strictly controlled based on a restrictive and prophylactic strategy in which each blood component was transfused separately according to its respective indication^35^. Blood salvage and auto-transfusion were routinely used to reduce recipient exposure to allogeneic red blood cells^36, 37^. Immunosuppression and hepatitis B virus prophylaxis were performed according to the standardized protocol, as described previously^38^.

### Statistical analysis

The primary outcome was post-transplant graft failure (death or retransplantation). The secondary outcome was post-transplant overall death. Survival analysis was performed using the Cox model, and the results were described using hazard ratios (HR) with 95% confidence interval. Backward stepwise selection was performed for multivariable analysis with p < 0.05 for inclusion and p > 0.10 for removal. Subgroup analysis was performed within the recipients who did not have hepatocellular carcinoma (HCC) to remove the bias from HCC-related graft failure because our recent research demonstrated that the risk of post-transplant HCC recurrence is greater in recipients of grafts from male donors^38^. Furthermore, for graft failure occurred during the early postoperative period (90 days), early allograft failure simplified estimation (EASE) score, which is the indicator for quantifying the early post-operative failure risk, was calculated compared between two groups^39, 40^. Regarding estrogen receptor analysis, the multivariable Cox model was used to test whether estrogen content of donor liver is associated with graft failure risk while the independent variables which were significant during the multivariable analysis for graft failure were included. Because the largest part of graft failure occurred during the early post-transplant period, we performed the analysis for the first 6 months post-transplantation. The continuous variables were described as median with interquartile range and analyzed using the Mann–Whitney U-test. The categorical variables were expressed as frequency (%) and analyzed using χ^2^ test or Fisher's exact test. All reported p-values were two-sided, and p < 0.05 was considered significant. Data were analyzed using SPSS 19.0 (SPSS Inc, Chicago, IL, US).

## Results

The indications for transplantation in the 780 recipients were as follows: HCC arising from viral hepatitis (n = 401; hepatitis B, n = 367; hepatitis C, n = 32; and hepatitis B and C, n = 2), alcoholic hepatitis (n = 16), or unknown origin (n = 18); liver failure secondary to viral hepatitis (n = 216; hepatitis A, n = 7; hepatitis B, n = 184; hepatitis C, n = 21; hepatitis B and C, n = 3; and hepatitis A and B, n = 1), alcoholic hepatitis (n = 51), biliary obstruction (n = 14), metabolic disease (n = 5), or unknown origin (n = 17); autoimmune hepatitis (n = 16); toxic hepatitis (n = 13); Budd-Chiari syndrome (n = 7); and liver tumors other than HCC (n = 6). There were 207 male recipients of grafts from female donors. Other 573 recipients consisted of 59 female recipients with female donors, 104 female recipients with male donors, and 410 male recipients with male donors. Clinical data of male recipients with female donors and other recipients are described in Table 1 (≤ 40 years non-macrosteatotic donors and ≤ 40 years macrosteatotic donors), Table 2 (> 40 years non-macrosteatotic donors and > 40 years macrosteatotic donors). There were no extremely small grafts with < 0.6% graft-to-recipient weight ratio.

**Table 1:** Comparison of clinical data of recipients with grafts from donors ≤ 40 years.

Variables	Non-macrosteatotic grafts		Macrosteatotic grafts	
	Other combinations (n = 236)	Female to male (n = 71)	Other combinations (n = 234)	Female to male (n = 52)
Graft factors				
Donor age (years)	25 (21–30)	28 (23–33)^##^	26 (23–32)	27 (21–32)
ABO blood type incompatible donor	36 (15.3)	7 (9.9)	27 (11.5)	9 (17.3)
Graft ischemia time (min)*	121 (104–142)	135 (103–168)^#^	121 (103–140)	130 (104–155)
Graft-to-recipient weight ratio < 0.8%**	24 (10.2)	17 (23.9)^##^	6 (2.6)	16 (30.8)^##^
Laparoscopic procurement	18 (7.6)	8 (11.3)	16 (6.8)	3 (5.8)
Hepatic inflow occlusion***				
0 round	131 (55.5)	47 (66.2)	129 (55.1)	34 (65.4)
1–2 rounds	56 (23.7)	17 (23.9)	50 (21.4)	12 (23.1)
≥ 3 rounds	49 (20.8)	7 (9.9)	55 (23.5)	6 (11.5)
Recipient factors				
Age (years)	53 (50–57)	52 (46–58)	54 (50–59)	54 (47–60)
Body mass index (kg/m^2^)	24.3 (22.2–26.6)	24.5 (22.1–26.4)	24.1 (22.1–26.1)	24.7 (21.7–26.6)
Hypertension	21 (8.9)	8 (11.3)	33 (14.1)	6 (11.5)
Diabetes	76 (32.2)	19 (26.8)	85 (36.3)	22 (42.3)
Hepatitis B virus prophylaxis era				
Monoprophylaxis (2002–2007)	20 (8.5)	6 (8.5)	17 (7.3)	5 (9.6)
Entecavir (2008–2011)	96 (40.7)	30 (42.3)	111 (47.4)	27 (51.9)
Entecavir (2011–2014)	120 (50.8)	35 (49.3)	106 (45.3)	20 (38.5)
Non-viral etiology	44 (18.6)	13 (18.3)	50 (21.4)	15 (28.8)
Hepatocellular carcinoma				
None	102 (43.2)	29 (40.8)	96 (41.0)	23 (44.2)
Within the Milan criteria	86 (36.4)	27 (38.0)	90 (38.5)	19 (36.5)
Beyond the Milan criteria	48 (20.3)	15 (21.1)	48 (20.5)	10 (19.2)
MELD score	15 (11–20)	15 (10–23)	14 (10–20)	13 (9–17)
Neutrophil-to-lymphocyte ratio	2.2 (1.4–3.7)	2.4 (1.7–4.9)	2.4 (1.4–4.7)	2.2 (1.4–4.0)
Albumin (g/dL)	3.2 (2.7–3.6)	3.2 (2.7–3.7)	3.1(2.8–3.6)	3.1 (2.7–3.7)
Platelet count (× 10^9^/L)	63 (41–92)	69 (45–102)	62 (44–99)	68 (49–102)
Refractory ascites	52 (22.0)	19 (26.8)	54 (23.1)	11 (21.2)
Hepatic encephalopathy				
None	193 (81.8)	58 (81.7)	185 (79.1)	43 (82.7)
Grade I–II	35 (14.8)	9 (12.7)	38 (16.2)	5 (9.8)
Grade III–IV	8 (3.4)	4 (5.6)	11 (4.7)	4 (7.7)
Preoperative continuous renal replacement	9 (3.8)	3 (4.2)	7 (3.0)	3 (5.8)
Operative time (min)	538 (494–623)	586 (509–648)^#^	579 (512–644)	558 (489–656)
Perioperative transfusion****				
Packed red blood cells (units)	4 (2–8)	4 (0–11)	4 (2–8)	2 (0–7)
Apheresis platelets (units)*****	1.3 (0–3.3)	1.0 (0–3.0)	1.3 (0–3.4)	1.0 (0–3.7)
Fresh frozen plasma (units)	2 (0–6)	5 (2–10)^##^	3 (0–8)	4 (2–6)
Cryoprecipitate (units)	3 (0–6)	3 (0–9)	6 (0–6)	3 (0–8)

Data are presented as frequency (%) or median (25th percentile, 75th percentile). *Cold ischemia time plus warm ischemia time **There were no recipients with graft-to-recipient weight ratio < 0.6% ***During each round of IHIO, the hepatic artery and the portal vein were clamped for 15 minutes and unclamped for 5 minutes, in general ****During surgery and within 2 weeks after surgery *****One unit of apheresis platelets was considered to be equivalent to 6 units of whole-blood platelets. ^#^p < 0.05, ^##^p < 0.01.

**Table 2:** Comparison of clinical data of recipients with grafts from donors > 40 years.

Variables	Non-macrosteatotic grafts		Macrosteatotic grafts	
	Other combinations (n = 52)	Female to male (n = 42)	Other combinations (n = 51)	Female to male (n = 42)
Graft factors				
Donor age (years)	48 (42–52)	45 (42–48)	48 (44–53)	51 (45–55)
ABO blood type incompatible donor	6 (11.5)	4 (9.5)	5 (9.8)	12 (28.6)^#^
Graft ischemia time (min)*	119 (103–139)	127 (108–163)	117 (101–135)	126 (102–146)
Graft-to-recipient weight ratio < 0.8%**	5 (9.6)	6 (14.3)	3 (5.9)	7 (16.7)
Laparoscopic procurement	1 (1.9)	1 (2.4)	0	2 (4.8)
Hepatic inflow occlusion***				
0 round	23 (44.2)	21 (50.0)	28 (54.9)	17 (40.5)
1–2 rounds	14 (26.9)	16 (38.1)	12 (23.5)	13 (31.0)
≥ 3 rounds	15 (28.8)	5 (11.9)	11 (21.6)	12 (28.6)
Recipient factors				
Age (years)	51 (43–57)	48 (46–53)	52 (43–62)	51 (44–57)
Body mass index (kg/m^2^)	23.7 (21.3–26.1)	24.0 (22.2–25.7)	24.5 (22.1–26.6)	23.9 (21.2–25.7)
Hypertension	5 (9.6)	4 (9.5)	7 (13.7)	9 (21.4)
Diabetes	16 (30.8)	18 (42.9)	25 (49.0)	16 (38.1)
Hepatitis B virus prophylaxis era				
Monoprophylaxis (2002–2007)	2 (3.8)	6 (14.3)	4 (7.8)	2 (4.8)
Entecavir (2008–2011)	21 (40.4)	17 (40.5)	22 (43.1)	11 (26.2)
Entecavir (2011–2014)	29 (55.8)	19 (45.2)	25 (49.0)	29 (69.0)
Non-viral etiology	14 (26.9)	5 (11.9)^#^	16 (31.4)	6 (14.3)
Hepatocellular carcinoma				
None	34 (65.4)	18 (42.9)^#^	27 (52.9)	16 (38.1)^#^
Within the Milan criteria	15 (28.8)	14 (33.3)	20 (39.2)	16 (38.1)
Beyond the Milan criteria	3 (5.8)	10 (23.8)	4 (7.8)	10 (23.8)
MELD score	17 (10–34)	15 (11–28)	16 (12–33)	14 (10–22)
Neutrophil-to-lymphocyte ratio	3.4 (2.2–6.0)	2.9 (1.7–6.3)	2.6 (1.5–6.1)	2.6 (1.7–4.1)
Albumin (g/dL)	3.1 (2.7–3.7)	3.0 (2.7–4.1)	3.1 (2.6–3.6)	3.3 (2.9–3.9)
Platelet count (× 10^9^/L)	85 (64–125)	58 (38–84)	71 (46–119)	67 (39–133)
Refractory ascites	12 (23.1)	12 (28.6)	16 (31.4)	6 (14.3)
Hepatic encephalopathy				
None	39 (75.0)	35 (83.3)	41 (80.4)	34 (81.0)
Grade I–II	8 (15.4)	6 (14.3)	7 (13.7)	5 (11.9)
Grade III–IV	5 (9.6)	1 (2.4)	3 (5.9)	3 (7.1)
Preoperative continuous renal replacement	3 (5.8)	2 (4.8)	2 (3.9)	1 (2.4)
Operative time (min)	547 (489–606)	568 (530–649)^#^	556 (495–603)	594 (517–643)^#^
Perioperative transfusion****				
Packed red blood cells (units)	4 (2–8)	3 (1–11)	6 (3–11)	6 (2–10)
Apheresis platelets (units)*****	1.2 (0–2.7)	2.0 (0–4.2)	2.0 (0–3.7)	1.3 (0–4.0)
Fresh frozen plasma (units)	6 (2–7)	6 (2–11)	4 (2–8)	5 (2–8)
Cryoprecipitate (units)	6 (0–9)	6 (0–8)	6 (0–6)	0 (0–6)^#^

Data are presented as frequency (%) or median (25th percentile, 75th percentile). *Cold ischemia time plus warm ischemia time **There were no recipients with graft-to-recipient weight ratio < 0.6% ***During each round of IHIO, the hepatic artery and the portal vein were clamped for 15 minutes and unclamped for 5 minutes, in general ****During surgery and within 2 weeks after surgery *****One unit of apheresis platelets was considered to be equivalent to 6 units of whole-blood platelets. ^#^p < 0.05, ^##^p < 0.01.

**Figure 1:**
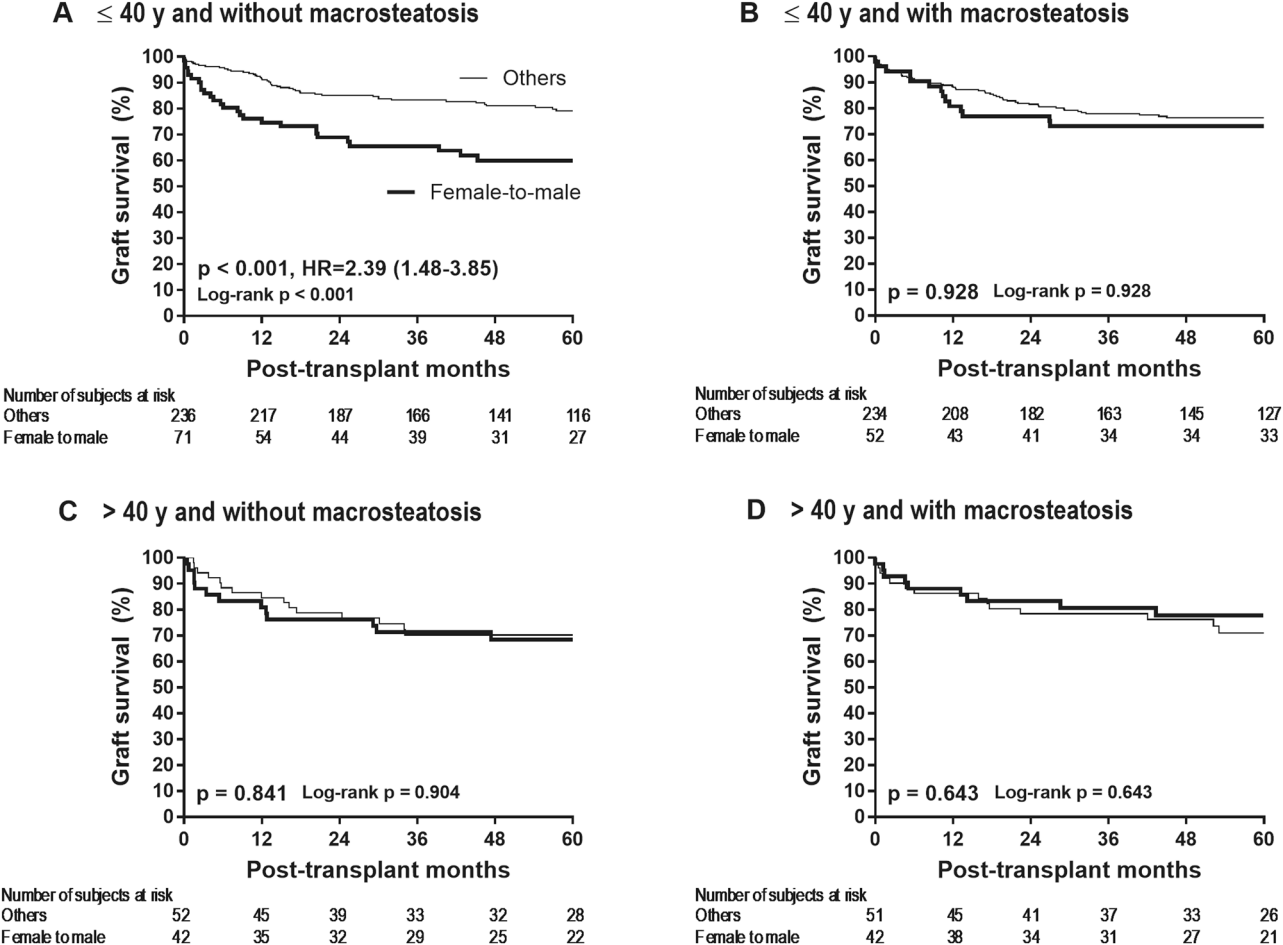
Graft failure risk according to donor/recipient sex within each of the four subgroups stratified by donor age and macrosteatosis.

The median follow-up time was 35 months with interquartile range of 20 to 60 months. Within the subgroup of recipients with ≤ 40 years non-macrosteatotic donors (Fig. 1A), graft failure risk was significantly greater in female-to-male LT than in other LTs (HR 2.39 [1.48–3.85], p < 0.001). The probability of graft failure after 1, 2, and 5 years post-transplantation was 25.4% (16.8%–37.2%), 31.2% (21.7%–43.4%), and 40.1% (29.36%–53.1%), respectively, in female-to-male LT and 8.5% (5.6%–12.8%), 14.9% (10.9%–20.1%), and 20.9% (16.0%–27.0%), respectively, in other LTs. In particular, there were no recipients with < 0.6% graft-to-recipient weight ratio. As shown in Table 3, the results of multivariable analysis demonstrated that female-to-male donation is an independent risk factor for graft failure within the subgroup of recipients with ≤ 40 years non-macrosteatotic donors (HR 2.03 [1.18–3.49], p = 0.011 and HR 1.88 [1.16–3.04], p = 0.011, respectively). Consistent effect sizes indicated the insignificant confounding effects from covariables and reliability of the dominant impact of donor/recipient sex. As shown in Fig. 1BCD, graft failure risk is not significantly different between female-to-male LT and other LTs when the donor was > 40 years or macrosteatotic (p > 0.60). In graft failure case occurred during the early postoperative period (90 days), EASE score was significantly lower in female-to-male LT than other LTs [Median (IQR) −2.2 (−3.2, 0.175) vs.  −1.5 (−2.9, 1), p = 0.036], indicating that graft quality was not a factor confoundg the result. In line with graft failure, death risk was also significantly greater in female-to-male LT than in other LTs only within the subgroup of recipients with ≤ 40 years non-macrosteatotic donors (HR 2.35 [1.35–4.09], p = 0.003), whereas it was comparable when the donor was > 40 years or macrosteatotic (p > 0.70), as shown in Supplementary Fig. 1. Supplementary Table 1 shows the most probable cause of death in female-to-male LTs and other LTs, respectively, within the recipients with ≤ 40 years non-macrosteatotic donors.

**Table 3:** Univariable and multivariable analysis for graft failure in recipients of non-macrosteatotic grafts from donors ≤ 40 years.

Variables	Univariable analysis		Multivariable analysis	
	Hazard ratio	p	Hazard ratio	p
Graft factors				
Female-to-male donation	2.22 (1.40–3.53)	0.001	1.88 (1.16–3.04)	0.011
Donor age (years)	1.01 (0.97–1.04)	0.709		
ABO blood type incompatible donor	0.68 (0.33–1.41)	0.301		
Graft ischemia time (min)*	1.00 (1.00–1.01)	0.326		
Graft-to-recipient weight ratio < 0.8%**	0.73 (0.35–1.52)	0.405		
Laparoscopic procurement	0.71 (0.26–1.94)	0.497		
Hepatic inflow occlusion***				
1–2 rounds (vs. 0 round)	0.50 (0.26–0.99)	0.046		
≥ 3 rounds (vs. 0 round)	1.08 (0.62–1.87)	0.785		
Recipient factors				
Age (years)	1.01 (0.98–1.04)	0.467		
Body mass index (kg/m^2^)	0.95 (0.89–1.01)	0.096	0.94 (0.88–0.99)	0.043
Hypertension	0.77 (0.33–1.76)	0.532		
Diabetes	1.28 (0.81–2.02)	0.295		
Hepatitis B virus prophylaxis era				
2008–2011 (vs. monoprophylaxis era)	0.74 (0.37–1.50)	0.406		
2011–2014 (vs. monoprophylaxis era)	0.84 (0.31–1.31)	0.225		
Non-viral etiology	0.86 (0.48–1.56)	0.626		
Hepatocellular carcinoma				
Within the Milan criteria (vs. none)	1.32 (0.76–2.29)	0.325	1.57 (0.85–2.91)	0.150
Beyond the Milan criteria (vs. none)	2.62 (1.52–4.53)	0.001	2.81 (1.54–5.13)	0.001
MELD score	1.01 (0.99–1.03)	0.438		
Neutrophil-to-lymphocyte ratio	1.02 (1.00–1.04)	0.040		
Albumin (g/dL)	0.96 (0.67–1.37)	0.801		
Platelet count (× 10^9^/L)	1.00 (1.00–1.01)	0.061		
Refractory ascites	0.65 (0.36–1.15)	0.138	0.46 (0.24–0.87)	0.017
Hepatic encephalopathy				
Grade I–II (vs. none)	0.69 (0.35–1.40)	0.306		
Grade III–IV (vs. none)	1.38 (0.50–3.80)	0.530		
Preoperative continuous renal replacement	2.49 (1.00–6.19)	0.049	5.12 (1.90–13.78)	0.001
Operative time (min)	1.00 (1.00–1.00)	0.024		
Perioperative transfusion****				
Packed red blood cells (units)	1.06 (1.04–1.07)	< 0.001	1.07 (1.05–1.08)	< 0.001
Apheresis platelets (units)*****	1.02 (1.01–1.02)	< 0.001		
Fresh frozen plasma (units)	1.01 (1.00–1.01)	0.048		
Cryoprecipitate (units)	1.04 (1.01–1.07)	0.012		

*Cold ischemia time plus warm ischemia time **There were no recipients with graft-to-recipient weight ratio < 0.6% ***During each round of IHIO, the hepatic artery and the portal vein were clamped for 15 minutes and unclamped for 5 minutes, in general ****During surgery and within 2 weeks after surgery *****One unit of apheresis platelets was considered to be equivalent to 6 units of whole-blood platelets.

**Figure 2:**
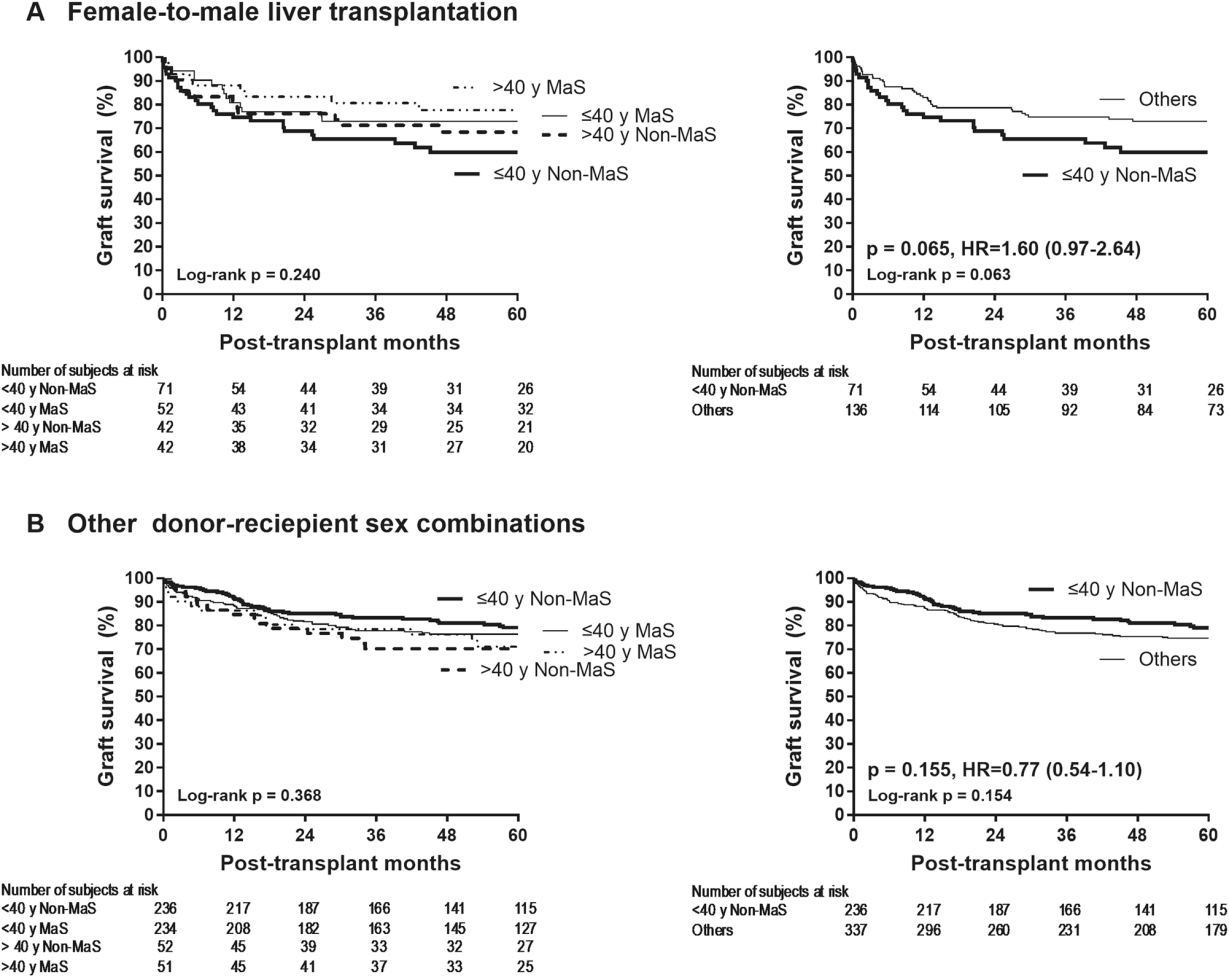
Graft failure risk according to donor age and macrosteatosis within each of the two subgroups stratified by donor/recipient sex.

Figure 2 shows the opposite effect of better graft quality (≤ 40 years and non-macrosteatosis) on graft failure risk in female-to-male LT and other LTs. In agreement with the general consensus, graft failure risk tended to be lower with better graft quality (≤ 40 years non-macrosteatotic donors vs. > 40 years or macrosteatotic donors) in other LTs than female-to-male LT (HR 0.77 [0.54–1.10], p = 0.155). In contrast, graft failure risk was insignificantly greater with better graft quality in female-to-male LT (HR 1.60 [0.97–2.64], p = 0.065).

Within the subgroup of non-HCC recipients, the gap in graft failure risk between female-to-male LT and other LTs when donors were ≤ 40 years and non-macrosteatotic became greater (HR 4.75 [2.02–11.21], p < 0.001) compared to the results for the whole cohort (Supplementary Fig. 2A). The probability of graft failure after 1, 2, and 5 years post-transplantation was 37.9% (23.1%–57.9%) in female-to-male LT (all graft failures occurred within 1 year) and 5.9% (2.7%–12.6%), 8.9% (4.7%–16.3%), and 9.9% (5.5%–17.7%), respectively, in other LTs. The causes of 11 graft failures after female-to-male LT were hepatic artery complication (n = 4), primary non-function (n = 2), biliary complication (n = 1), viral recurrence (n = 1), graft rejection (n = 1), and infectious complication (n = 2). When donors were > 40 years or macrosteatotic, there were no significant differences between female-to-male LT and other LTs (Supplementary Fig. 2BCD) in line with the results for the whole cohort. Supplementary Table 2 shows the most probable cause of graft failure in female-to-male LT and other LTs, respectively, within the non-HCC recipients with ≤ 40 years non-macrosteatotic donors.

**Figure 3:**
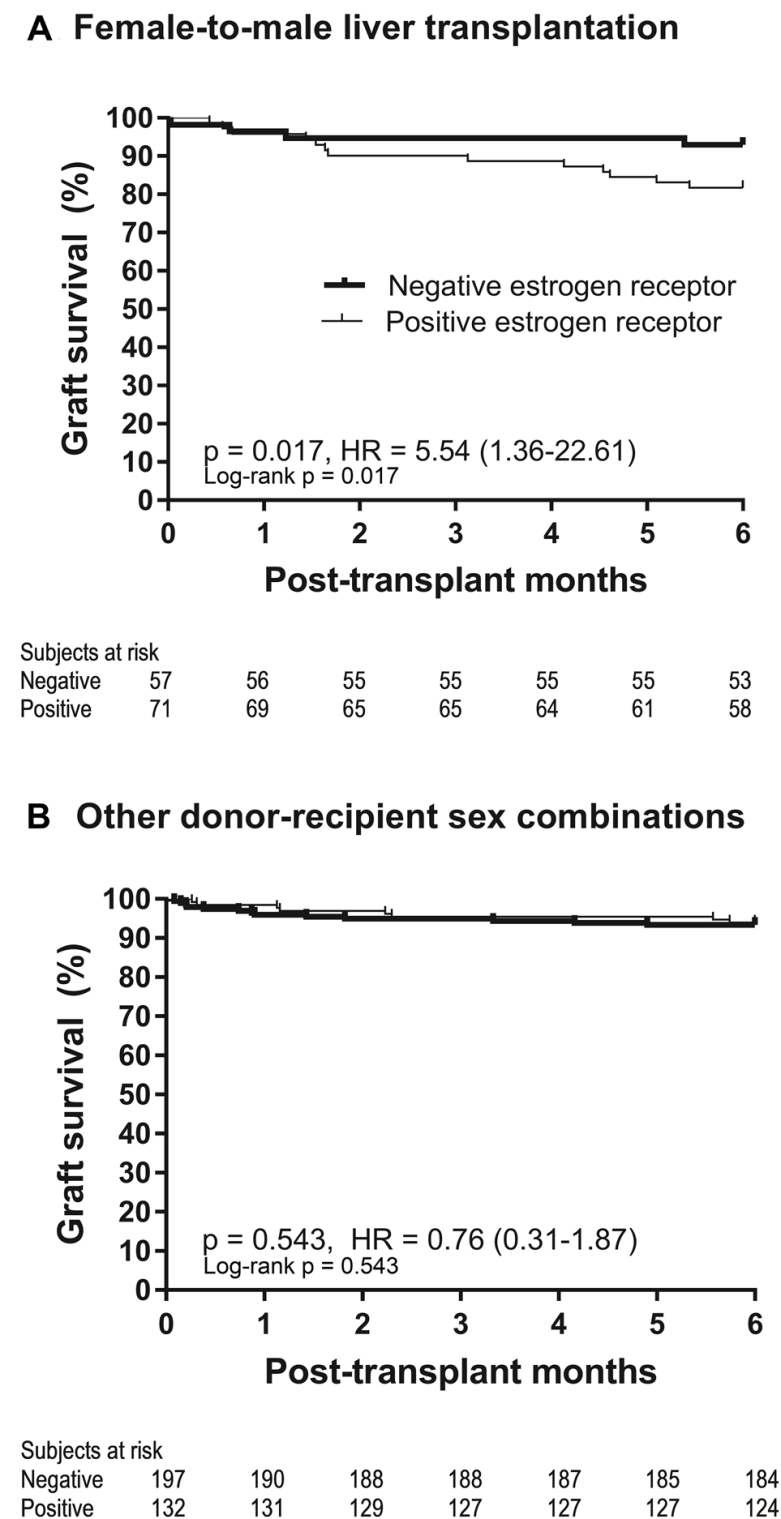
Post-transplant graft failure within 6 months according to the status of graft estrogen receptor.

As shown in Fig. 3 and Table 4, in the subgroup of patients who underwent female-to-male LT, the risk of graft failure within 6 months after transplantation was significantly greater in patients who received a graft positive at estrogen receptor (HR 5.54 [1.36–22.61], p = 0.017). In contrast, in the subgroup of patients who underwent other LTs than female-to-male LT, the risk of graft failure within 6 months was not significantly different (HR 0.76 [0.31–1.87], p = 0.543).

**Table 4:** Multivariable analysis testing the association of liver graft estrogen receptor status with graft failure risk in female-to-male liver transplantation.

Variables	Female-to-male		Other combinations	
	Hazard ratio	p	Hazard ratio	p
Positive graft estrogen receptor	5.54 (1.36–22.61)	0.017	0.76 (0.31–1.87)	0.543
Donor age > 40 years	1.01 (0.97–1.06)	0.663	1.04 (1.00–1.08)	0.071
Graft macrosteatosis > 5%	1.34 (0.42–4.23)	0.622	1.89 (0.75–4.80)	0.179
Body mass index (kg/m^2^)	1.01 (0.84–1.22)	0.909	1.00 (0.99–1.01)	0.869
Hepatocellular carcinoma				
Within the Milan criteria (vs. none)	0.39 (0.11–1.35)	0.137	1.15 (0.40–3.35)	0.796
Beyond the Milan criteria (vs. none)	0.20 (0.04–1.13)	0.069	1.05 (0.27–4.01)	0.946
Refractory ascites	0.07 (0.01–0.71)	0.024	1.07 (0.38–3.03)	0.897
Preoperative continuous renal replacement	0.32 (0.01–9.45)	0.506	1.44 (0.27–7.62)	0.671
Packed red blood cells (units)	1.14 (1.17–1.21)	< 0.001	1.01 (1.06–1.14)	< 0.001

Multivariable analysis included the variables that were significant in the multivariable model in Table 3 while donor age and graft macrosteatosis are forced in to the model.

## Discussion

Continuing from our previous work, demonstrating the greater tolerance of hepatic ischemia-reperfusion injury in females than in males only with age ≤ 40 years and no macrosteatosis^18^, the inferiority of female-to-male LT in graft failure, which is thought to result from the acute decrease in hepatic estrogen signaling and loss of hepatic protection capacity^6–8, 14^, was found only when the donor was ≤ 40 years and with no macrosteatosis. An exceptionally high graft failure risk of female-to-male LT, despite good graft quality and lower EASE score value, suggests the presence of an estrogen-related dominant contributor for graft failure that outweighs the effects of graft quality. Such high graft failure risk was not observed in female-to-male LT with > 40 years donors (decreased systemic estrogen secretion in the lack of ovarian reserve [infertility] or macrosteatotic donors (decreased hepatic estrogen receptor expression) despite worse graft quality^12–15, 18^, supporting the role of hepatic sex hormonal mismatch in the inferiority of female-to-male LT. When donor was > 40 years or macrosteatotic, the inferiority of female-to-male LT was abrogated and graft failure risk was comparable between female-to-male LT and other LTs, suggesting that anatomical size mismatch (e.g. small graft size or insufficient graft-to-recipient weight ratio) is not the reason of the inferiority of female-to-male LT, being in agreement with our recent research^11^. Overall, our data suggest the presence of dominant contributors for graft failure other than graft quality/quantity and supports the role of hepatic estrogen signaling mismatch in greater graft failure after female-to-male LT. Also, our data suggest the risk of female-to-male LT can be changed by modification of donor/recipient hepatic estrogen signaling.

Post-transplant liver graft failure has been known to be more prevalent in male recipients of female donors compared to other donor-recipient sex combinations and hepatic estrogen signaling is considered to play an important role in this sex difference^12–15^. Hepatic estrogen signaling is positively correlated with hepatic protection capacity^10, 12, 16–18^. A previous study in mice demonstrated that 100% of female mice survived after 70% hepatic inflow occlusion for 45 min, whereas all male mice died within 5 d with greater initial hepatocyte injury^10^. In the same study, ovariectomy or estrogen antagonist administration increased hepatic ischemia–reperfusion injury, reduced regenerative capacity, and increased mortality, whereas administration of 17β-estradiol improved recovery^10^. In rats, translocation of estrogen receptors from the cytosol to nucleus and activation of cell signaling occurred after liver injury to stimulate the healing process^41–43^. Our recent study of healthy living liver donors has also demonstrated that the tolerance of hepatic ischemia–reperfusion injury following intermittent hepatic inflow occlusion was greater in females than in males^18^. Thus, it has been hypothesized that acute decrease in hepatic estrogen signaling (including decreased hepatic estrogen receptor content) of the transplanted female liver after the exposure to male hormonal milieu impairs the recovery of female grafts^6–8^. The abrogation of the inferiority of female-to-male LT when the donor was > 40 years^22–25^ or with macrosteatosis^16, 26, 27^ supports the relationship because experiencing some degree of defeminization with lower hepatic estrogen signaling prior to transplantation may mitigate the impact of male hormonal milieu after transplantation.

The current study suggests that the insignificance of female-to-male LT in few previous articles were attributable to the lack of consideration of hepatic estrogen signaling or pretransplant liver graft defeminization according to reproductive aging and macrosteatosis^44–46^, along with the significant multicollinearity between donor sex and donor characteristics like weight and hight as indicated previously^11^. Also, biasing effects of HCC-related graft failure might have contributed because the impact of donor gender on HCC-unrelated graft failure and HCC-related graft failure is different based on our and other data^38^. In other words, analyzing the interaction of donor/recipient sex with donor age and macrosteatosis, instead of simply incorporating those variables in multivariable model, on HCC-related/unrelated graft failure may help improve research quality and derive more consistent results regarding the impact of female-to-male donation. This is important because there is a possibility that donor selection process may ultimately improve post-transplant clinical outcome if donor sex is appropriately taken into account while the greater graft failure risk in female-to-male LT has been being reported repeatedly. This is particularly relevant in living donor LT because multiple donation candidates can be obtained in some cases^44^. On the other hand, our data suggested that female-to-male LT is not inferior in all cases; thus, variables interacting with donor sex (e.g. donor age and macrosteatosis in the current study) needs to be further elucidated to minimize the risk subgroup. Also, our data suggest that pre/post-transplant hepatic estrogen conditioning to prevent acute defeminization of the graft helps maintain the hepatic protection capacity and prevent graft failure. More scientific data are warranted to incorporate donor sex and hepatic estrogen signaling into LT process.

In our analysis, we observed that the significant difference in graft failure between female-to-male LT and other LTs was only found with non-macrosteatotic donors ≤ 40 years. The absence of a correlation between EASE score and female-to-male sex match further supports that the physiopathologic mechanism underlying the inferiority of the female-to-male LT is not dependent on graft quality. An unexpected finding was a significant protective effect of recurrent ascites in the subgroup ‘female-to-male LT'. Despite the initial counterintuitive nature of this finding, potential confounding factors and complex underlying mechanisms need to be considered. The presence of recurrent ascites may trigger closer monitoring, early interventions, and more intensive management strategies, leading to improved graft outcomes. These interventions may include adjustments in immunosuppressive regimens, fluid balance optimization, and close surveillance for complications. An interesting finding was the association between female-to-male LT and sepsis-induced graft failure. Because the number of event was too small with the lack of statistical power, the current study cannot draw a conclusion for this finding warranting further investigation.﻿

Despite the retrospective design, our study presented several strengths for generation of robust data. First, this study included a homogeneous living donor liver transplant population. All recipients received right hemi-liver graft without variation. Most recipients underwent elective surgery and were in stable condition without acute deterioration. Accordingly, thorough perioperative anesthetic and surgical care could be performed strictly based on the institutional standardized protocols. Second, only three patients were lost to follow-up, and graft failure was defined as a clear objective outcome; thus, the data regarding the time-to-graft failure were highly reliable. Another advantage of this study was the routine use of Oil Red O staining to examine liver parenchyma. Conventional hematoxylin and eosin staining alone tends to underestimate the degree of steatosis^45^.

This study has several limitations as well. First, due to the retrospective nature of the study design, we were unable to exclude the possibility of bias from unmeasured variables, although we performed adjustment of all established contributors by subgroup analysis and multivariable analysis. Second, mechanisms underlying the sex differences in post-transplant graft failure as well as its change according to donor age and macrosteatosis have not yet been confirmed although the involvement of hepatic estrogen signaling can be assumed based on results of previous and the current studies. Third, in contrast to living donors, deceased donors are frequently attended with multiorgan dysfunction, unstable hemodynamics, vasoactive drug use, lack of sufficient hepatic inflow, and prolonged graft ischemia time^46^. Thus, it is unclear that the effects of donor age and macrosteatosis are observed in the same way in deceased donor LT because more various factors, aside from donor age and macrosteatosis, may affect hepatic estrogen signaling.

In the current study, we found that the greater graft failure risk of female-to-male LT was abrogated when the donor was > 40 years or with macrosteatosis, which is known to be related to the decrease in hepatic estrogen signaling. Therefore, our findings shed light on the importance of hepatic estrogen signaling mismatch between donor and recipient to post-transplant graft failure and suggest that the strategies to avoid abrupt decrease in hepatic estrogen signaling prevent graft failure in female-to-male LT.

### Supplementary Information

Supplementary Figure 1.

Supplementary Figure 2.

Supplementary Table 1.

## Data Availability

The datasets generated during and analyzed during the current study are available from the corresponding author on reasonable request.

## Abbreviations

EASE: Early allograft failure simplified estimation
HCC: Hepatocellular carcinoma
HR: Hazard ratio
LT: Liver transplantation
