# Supplementary figures and images for: Abrogation of greater graft failure risk of female-to-male liver transplantation with donors older than 40 years or graft macrosteatosis greater than 5%
Source: Sci Rep. 2023 Aug 9;13:12914. doi: 10.1038/s41598-023-38113-w (PMC10412610; doi:10.1038/s41598-023-38113-w)

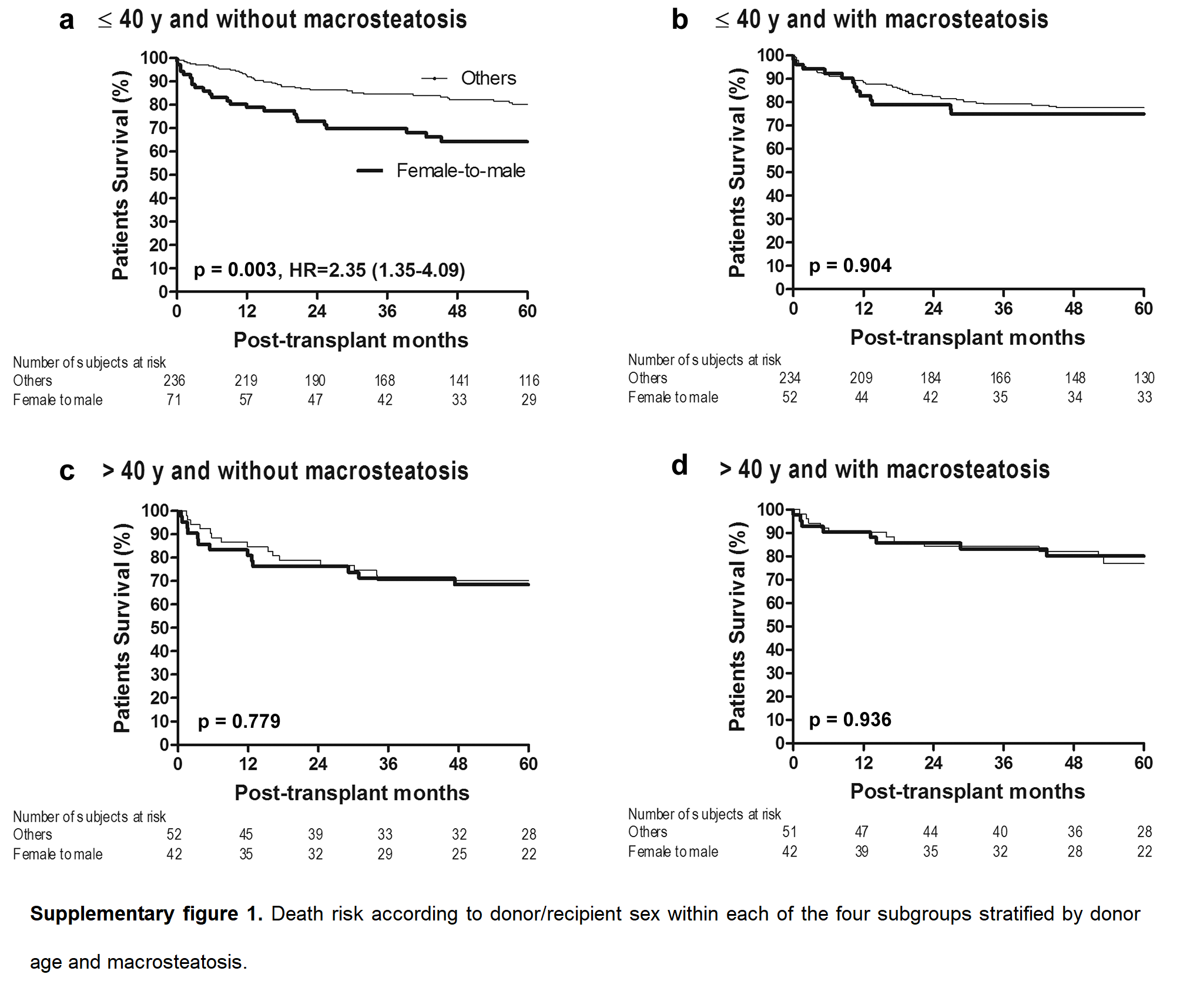

Supplement: Supplementary file 1 — Supplementary Figure 1. [file 41598_2023_38113_MOESM1_ESM.tif]

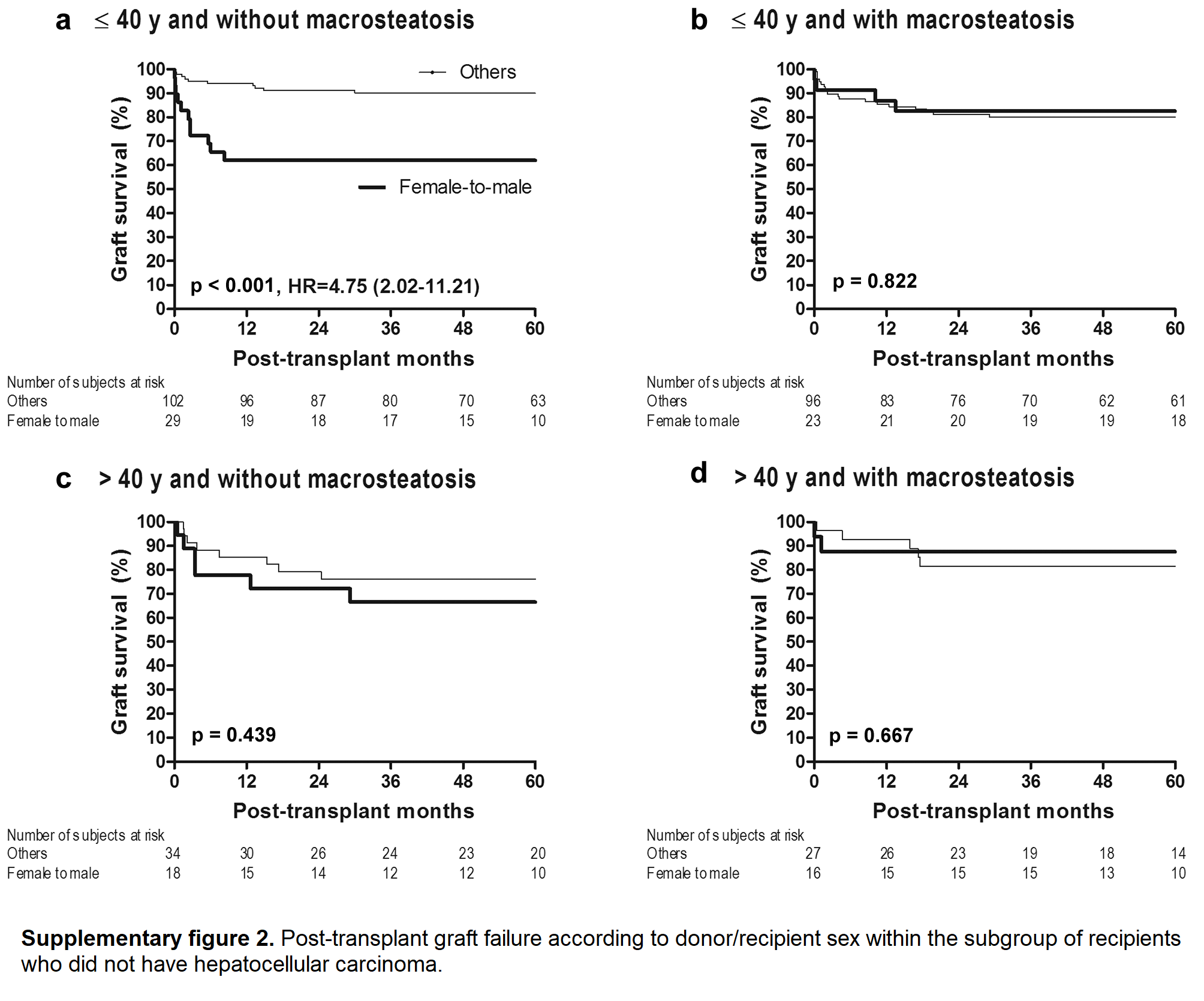

Supplement: Supplementary file 2 — Supplementary Figure 2. [file 41598_2023_38113_MOESM2_ESM.tif]
