# Supplementary material for: Abrogation of greater graft failure risk of female-to-male liver transplantation with donors older than 40 years or graft macrosteatosis greater than 5%
Source: Sci Rep. 2023 Aug 9;13:12914. doi: 10.1038/s41598-023-38113-w (PMC10412610; doi:10.1038/s41598-023-38113-w)
Supplement: Supplementary file 3 — Supplementary Table 1. [file 41598_2023_38113_MOESM3_ESM.docx]

**Supplementary Table 1.** Cause of death and graft failure according to the effect of female-to-male match in recipients with age >40 years without macrosteatosis

| **Cause of death^*^** | | **Other combinations**  **( 33 / 236 )** | **Female to male**  **( 20 / 71 )** | **P-value** |
| --- | --- | --- | --- | --- |
| **Malignancy** |  |  |  | 0.051 |
|  | HCC recurrence | 15 | 5 |  |
|  | HCC multiple metastasis | 2 | 1 |  |
|  | Other malignancy | 2 | 0 |  |
| **Graft failure** |  |  |  | 0.141 |
|  | Primary non-function | 0 | 1 |  |
|  | Vascular complication | 1 | 3 |  |
|  | Biliary complication | 2 | 2 |  |
|  | Rejection | 1 | 1 |  |
|  | Unknown | 3 | 1 |  |
| **Other cause** |  |  |  | 0.471 |
|  | Sepsis | 1 | 3 |  |
|  | Cardiovascular disease | 2 | 1 |  |
|  | Non-liver Organ failure | 0 | 1 |  |
|  | Surgical complication | 1 | 0 |  |
|  | PTLD | 0 | 1 |  |
|  | Suiside | 1 | 0 |  |
|  | Unknown | 2 | 0 |  |
|  |  |  |  |  |
|  | **Cause of graft failure^#^** | **Other combinations**  **( 10 / 102 )** | **Female to male**  **( 11 / 29 )** | **P-value** |
| **Liver related causes** |  |  |  | 0.195 |
|  | Primary non-function | 1 | 3 |  |
|  | Rejection | 1 | 0 |  |
|  | PTLD | 0 | 1 |  |
|  | Vascular complication | 2 | 2 |  |
|  | Biliary complication | 1 | 2 |  |
| **Other causes** |  |  |  | 0.007 |
|  | Sepsis | 0 | 2 |  |
|  | Other malignancy | 3 | 0 |  |
|  | Unknown | 2 | 1 |  |

**^*^** Cause of death according to the effect of female-to-male match in recipients with age >40 years without macrosteatosis (population in Fig. 2a), **^#^**Cause of Graft failure according to the effect of female-to-male match in recipients with age >40 years without macrosteatosis within the subgroup of recipients who did not have hepatocellular carcinoma (Population in Fig. 4 a)
